# Electronic specific heats for amorphous and crystallized alloys

**DOI:** 10.1186/s40064-016-2335-x

**Published:** 2016-05-23

**Authors:** Long Hou, Jinyong Mo, Qingling Liu, Haishun Liu, Weiming Yang, Baolong Shen

**Affiliations:** School of Sciences, School of Mechanics and Civil Engineering, State Key Laboratory for Geomechanics and Deep Underground Engineering, China University of Mining and Technology, Xuzhou, 221116 China; School of Materials Science and Engineering, Southeast University, Nanjing, 211189 China

**Keywords:** Amorphous alloy, Low temperature specific heat, Electronic density of states, Localized atoms

## Abstract

The low temperature specific heats of (Fe_0.5_Co_0.5_)_72_B_20_Si_4_Nb_4_ amorphous and crystallized alloys are measured and analyzed from 1.4 to 110 K. Specific heats can be well fitted by electronic and phonon contribution terms. It is found that the electronic contribution term in specific heat for amorphous alloy is larger than that for crystallized one, and this phenomenon has been interpreted in detail. The research shows that the electronic density of states at the Fermi level and the localized loose “rattler” atoms in oversized cage structure may make contributions to the enhancement of electronic specific heat coefficient *γ*, and result in a larger electronic contribution term. This study is significant for further understanding the structure–property relationship for amorphous alloys at low temperature.

## Background

As a new kind of material, amorphous alloy is known for its promising mechanical (Yang et al. 2014a, b; Dun et al. 2014), magnetic (Liu et al. 2014; Xiang et al. 2014), and chemical (Wang et al. 2012) properties compared to its corresponding crystalline counterpart, and the great differences in properties between the two states are considered to be connected with their ordered or disordered microstructure. Meanwhile, recent study also has revealed that the short- or medium-range order exists at the atomic scale in amorphous alloy (Wang et al. 2008). For the investigation of this interesting atomic structural characteristics and the corresponding mechanism, the low temperature specific heat is considered to be one of the most effective parameters, which is closely linked to the phase transformation (Mikla and Mikla 2009; Xiang et al. 2015), low-energy excitation (Huang 1988; Burin 1995), atomic vibrations (Li et al. 2008), and electronic movement of the solids (Machida and Ichioka 2008). It is known that amorphous alloys can be transformed into crystallized ones by annealing, and obvious changes are induced in the microstructure, accompanied with the variation of the specific heat. So far, for crystallized alloy, the low temperature specific heat can be explained satisfactorily using Debye model, an important theory in solid state physics. On the contrary, for amorphous alloy, although some achievements have been made recently (Blázquez et al. 2008; Kroeger et al. 1984; Kanomata et al. 2008; Grace and Anderson 1989), it still remains a puzzle that the specific heat obtained from experiment is usually a little larger than that from theoretical calculation at low temperature, moreover, when *T* > 1 K, the phonon specific heat, *C*_phonon_ still deviates from the expected *T*^3^ dependence, presenting a broad maximum in *C*_phonon_/*T*^3^, which is called the “boson peak” (BP) (Zheng 1987; Shintani and Tanaka 2008). Therefore, studying the intrinsic mechanisms of low temperature specific heats for both amorphous and crystallized alloys and exploring the relationship between them are becoming more and more necessary. Zhou et al. (2006) reported that the phonon specific heat at 1.8–154 K can be fitted using Debye model and Einstein oscillators model; Wang et al. (2011) studied the low temperature specific heat of Cu_60_Zr_20_Hf_10_Ti_10_ alloy and fitted the specific heats of amorphous and crystallized alloys linearly; Our previous work (Hou et al. 2015) also suggested that the low temperature specific heat can be fitted by electronic and phonon contribution terms. However, these studies mainly focus on the effect of crystallization on phonon specific heat, which induces the change of BP, while the study of crystallization on electronic specific heats for amorphous alloys is very little. At the same time, it has also been concluded that the electronic specific heat is closely related to the structural and mechanical properties (Yang et al. 2013; Yu et al. 2010). Thus, studying the effect of crystallization on the electronic specific heat has important scientific significance, it may disclose the change of electronic density of states and how this change affects the structure of amorphous alloy.

In this paper, from the perspective of electronic specific heat, the electronic contribution term in specific heat for (Fe_0.5_Co_0.5_)_72_B_20_Si_4_Nb_4_ amorphous alloy with the high glass-forming ability (GFA) and a highly random packed microstructure is discussed at low temperature. The result is expected to shed light on the disordered atomic structure in amorphous alloys at low temperature.

## Experimental methods

Multi-component Fe-Co-B-Si-Nb alloy ingots with composition of (Fe_0.5_Co_0.5_)_72_B_20_Si_4_Nb_4_ are prepared by arc melting the mixtures of Fe (99.99 mass %), Co (99.99 mass %), Nb (99.99 mass %) metals, together with B (99.50 mass %) and Si (99.99 mass %) crystals in a highly purified argon atmosphere. The alloy compositions represent nominal atomic percentages. The cast bulk (Fe_0.5_Co_0.5_)_72_B_20_Si_4_Nb_4_ amorphous alloy rods are fabricated by a copper-mold casting method (Yang et al. 2013). In the mold casting method, the master alloy is melted in a quartz crucible using an induction coil and pushed thereafter in a copper-mold by applying an ejection pressure of about 0.5 atm. To anneal the sample, the cast rod is cut to pieces 10 mm in length and placed in silica tube. The silica tube is sealed after evacuated and put into the furnace in vacuum, annealed at 923 K for 4 h, and then quenched in water. The annealing temperature is higher than the crystallization temperature (*T*_x_ = 857 K).

Amorphous and crystallized structures are examined by X-ray diffraction (XRD) using a RINT 2000 diffractometer with Cu K*a* radiation at 40 kV. The low temperature specific heats *C*_p_ for (Fe_0.5_Co_0.5_)_72_B_20_Si_4_Nb_4_ alloys in cast and crystallized states are measured using the physical property measurement system (PPMS) from quantum design system (Model-9) from 1.4 to 110 K. The final data are obtained by averaging the five experimental results. The relative error for the specific heat measurements is less than 2 %.

## Results and discussion

**Fig. 1:**
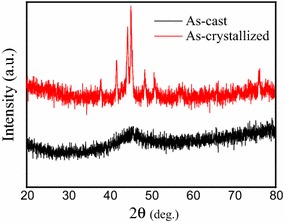
XRD patterns of (Fe_0.5_Co_0.5_)_72_B_20_Si_4_Nb_4_ alloys in the cast state and crystallized state

Figure 1 shows the XRD patterns of (Fe_0.5_Co_0.5_)_72_B_20_Si_4_Nb_4_ alloys in the cast state and crystallized state. Within the scope of the resolution of the XRD, the cast alloy shows a broad diffused peak without the crystallization crystal diffraction peak. This is a characteristic for fully amorphous alloy. After being annealed at 923 K for 4 h, the crystallized alloy shows many shap diffraction peaks, which indicates the crystallized state formed.

**Fig. 2:**
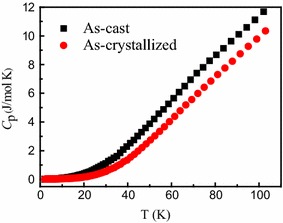
The *T*-dependent *C*_p_ for (Fe_0.5_Co_0.5_)_72_B_20_Si_4_Nb_4_ alloys in the cast state and crystallized state

Figure 2 presents *T*-dependent *C*_p_ for (Fe_0.5_Co_0.5_)_72_B_20_Si_4_Nb_4_ alloys in the cast state and crystallized state from 1.4 to 110 K. It can be seen that the *T*-dependent *C*_p_ curves for the samples in the cast state has larger specific heat than that in the crystallized state, and the *C*_p_ in these two states all increase with the temperature rising. In addition, the specific heat differences are not obvious up to 4 K in their total specific heat curves.

**Fig. 3:**
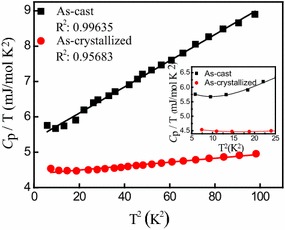
The *C*_p_ */T* versus *T* ^2^ for (Fe_0.5_Co_0.5_)_72_B_20_Si_4_Nb_4_ alloys in the cast state and crystallized state. The *solid lines* are linear square fits of the data. The *inset* is the enlarged plot for *T* ^2^ $\le$ 25 K^2^

**Fig. 4:**
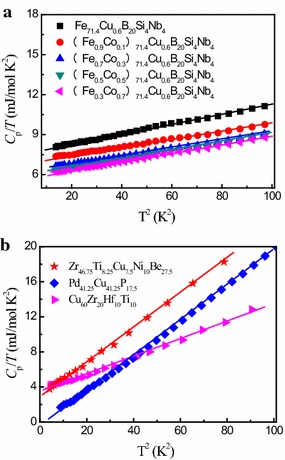
The *C*_p_ */T* versus *T* ^2^ for amorphous alloys. The *solid lines* are linear square fits of the data. The **a** data are taken from (Yang et al. 2014a, b) and **b** data are taken from (Wang et al. 2011; Yang et al. 2014b; Vasiliev et al. 2009)

Figure 3 shows the *C*_p_*/T* versus *T*^2^ plots for (Fe_0.5_Co_0.5_)_72_B_20_Si_4_Nb_4_ alloys in the cast state and crystallized state. In this figure, on one hand, it can be seen that the data points all fall apparently on straight lines over the range of 20 K^2^ ≤ *T*^2^ ≤ 100 K^2^ for (Fe_0.5_Co_0.5_)_72_B_20_Si_4_Nb_4_ amorphous alloy. We analyze the low temperature specific heat of (Fe_0.5_Co_0.5_)_72_B_20_Si_4_Nb_4_ amorphous alloy and fit it using a polynomial, and the arbitrary combinations of four terms (*A*, *T*, *T*^2^, and *T*^3^, where *A* is a constant) are considered in the fitting procedure. It is found that combination of *T* and *T*^3^ terms is a unique, reasonable, and accurate fit (correlation *R*^2^ = 0.99635), while the other terms are unreasonable fit for samples. For example, a polynomial fit to the specific heat of amorphous alloy with form of *C*_p_ = *A*_0_ + *A*_1_*T* + *A*_2_*T*^2^ + *A*_3_*T*^3^ produces negative values of the coefficients *A*_1_ and *A*_3_, which are obviously unreasonable in physics. Similar problem exists in fit by combination of *T*, *T*^2^, and *T*^3^. Fitting is also unacceptable by only one term of *T*^3^, which is in agreement with the previous report (Bai et al. 2002). Moreover, the combination of *T* and *T*^3^ terms obtained is applied in the fitting and compared with previous experimental data, as shown in Fig. 4a, b. So, it is reasonable to employ the fitting expression *C*_p_ = *γT* + *δT*^3^ (where *γ*, *δ* are constant, and *γ* = *A*_1_; *δ* = *A*_3_) with different *γ* and *δ* values, and Table 1 shows the fitting parameters of the specific heats. On the other hand, from Figs. 3 and 4, we can see that when *T*^2^ ≤ 20 K^2^, the experimental data all are slightly deviated from the linear fitting results, and represents a boson peak, respectively, in *C*_phonon_/*T*^3^ versus *T* curves, which has been reported in detail (Zheng 1987; Shintani and Tanaka 2008; Hou et al. 2015). The inset of Fig. 3 shows the enlarged plot for *T*^2^ ≤ 25 K^2^, and when *T*^2^ ≤ 20 K^2^, it shows an obvious curve. Thus, we study the specific heat data when *T* ≥ 4.5 K and the fitting expression is reasonable.

**Table 1:** Values of *γ*, *δ* and $\theta_{\text{D}}$ for alloys in the cast state and the crystallized (CR) state

Number	Samples	States	*γ* mJ/(mol K^2^)	*δ* mJ/(mol K^4^)	$\theta_{\text{D}}$/K
1	Zr_41.2_Ti_3.8_Cu_12.5_Ni_10_Be_22.5_^a^	Cast	3.030	0.101	268.120
		CR-805 K	1.830	0.053	331.500
2	Zr_52.5_Cu_17.9_Ni_14.6_Al_10_Ti_5_^b^	Cast	3.440	0.150	234.890
		CR-973 K	3.170	0.077	293.360
3	(Cu_50_Zr_50_)_90_Al_7_Gd_3_^c^	Cast	4.900	0.210	209.820
		CR-520 K	4.770	0.200	213.260
4	(Cu_50_Zr_50_)_96_Al_4_^d^	Cast	3.700	0.180	220.880
		CR-490 K	3.461	0.165	227.380
5	(Cu_50_Zr_50_)_92_Al_8_^e^	Cast	3.060	0.170	225.290
		CR-673 K	2.970	0.100	268.880
6	(Fe_0.5_Co_0.5_)_72_B_20_Si_4_Nb_4_^f^	Cast	5.366	0.037	374.540
		CR-923 K	4.385	0.006	686.830
7	Cu_50_Zr_50_^g^	Cast	4.300	0.129	246.830
		CR-765 K	2.740	0.120	252.840

^a^Zhang et al. (2001)

^b^Hou et al. (2015)

^c^Li et al. (2006, 2008)

^d,e^Li et al. (2008)

^f^This work

^g^Tang et al. (2005)

**Fig. 5:**
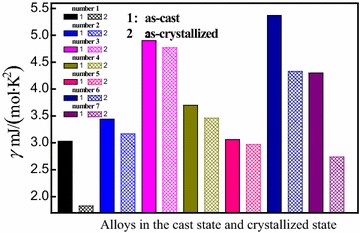
The changes of electronic specific heat coefficient *γ* for alloys in the cast state and crystallized state

From Table 1, one can see that *γ* value is changed from 5.366 to 4.385 mJ/(mol·K^2^) with annealing from cast to crystallized state for (Fe_0.5_Co_0.5_)_72_B_20_Si_4_Nb_4_ alloy. In order to explore whether this change is universal, we choose Zr_41.2_Ti_3.8_Cu_12.5_Ni_10_Be_22.5_ (Zhang et al. 2001), Zr_52.5_Cu_17.9_Ni_14.6_Al_10_Ti_5_ (Hou et al. 2015), (Cu_50_Zr_50_)_90_Al_7_Gd_3_ (Li et al. 2006, 2008), (Cu_50_Zr_50_)_96_Al_4_ (Li et al. 2008), (Cu_50_Zr_50_)_92_Al_8_ (Li et al. 2008) and Cu_50_Zr_50_ (Tang et al. 2005) alloys to obtain relevant parameters *γ*, *δ* by fitting their low temperature specific heats, and the corresponding Debye temperature $\theta_{\text{D}}$ is calculated by $\delta = \frac{{12\pi^{4} }}{{5\theta_{\text{D}} }}R$ (Huang 1988). They are also shown in Table 1. Meanwhile, Fig. 5 shows the changes of *γ* for alloys in the cast state and the crystallized state, in which the solid and cross-hatched histograms represent alloys in the cast state and the crystallized state, respectively. It clearly can be seen from Table 1 and Fig. 5 that *γ* value in crystallized state is smaller than that in the cast state.

The difference of *γ* values in amorphous and crystallized alloys stimulates us to think over the following questions: what is the relationship between specific heats for amorphous and crystallized alloys, and how does the microstructure change affect the specific heat? Thus, theoretical analysis on the similarities and differences of specific heat at low temperature in two states and the effects of crystallization are provided in the following part.

### Specific heats for crystallized alloys

For crystallized alloys, previous studies (Huang 1988; Luborsky 1989) have concluded that the specific heat $C_{\text{p}}^{1}$ at low temperature is comprised of an electronic term and a phonon term, which can be expressed as:

1 $$C_{\text{p}}^{1} = \gamma_{\text{c}} T + \delta_{\text{c}} T^{3}$$

where $\gamma_{\text{c}} = \frac{1}{3}\pi^{2} k_{B}^{2} N\left( {E_{F}^{0} } \right)$ is electronic specific heat coefficient, $\delta_{\text{c}} = \frac{{12\pi^{4} }}{5}R\left( {\frac{{k_{\text{B}} }}{{\hbar w_{\text{m}} }}} \right)^{3}$ is phonon specific heat coefficient, *k*_B_ is the Boltzmann constant, *R* is the gas constant, *w*_*m*_ is Debye-type vibrational frequency and $\hbar$ is reduced Planck’s constant. This theory is widely used for crystallized alloys, so relating discussion has not been performed in this study.

### Specific heats for amorphous alloys

Compared to crystallized alloys, although amorphous alloys do not have long-range atomic order, they do have pronounced short- or medium-range order at atomic scale, so we should consider their microstructure in disordered and some extent ordered arrangements simultaneously that may affect their specific heats.

To make this issue simpler, we consider the characteristic of some extent ordered microstructure. Like crystallized alloy, the low temperature specific heat *C*_p1_ for amorphous alloy can also be regarded as the contributions from the phonons and electrons, and amorphous alloy is also considered as a continuous elastic medium (Zhang et al. 2001), thus, according to the energy band theory (Huang 1988), the phonon contribution to specific heat can be expressed by the cubic Debye’s term *C*_D1_, i.e., *C*_D1_ = *δ*_1_*T*^3^, where $\delta_{1} = \frac{{12\pi^{4} }}{5}R\left( {\frac{{k_{\text{B}} }}{{\hbar w_{\text{m}} }}} \right)^{3}$ is phonon specific heat coefficient. For the electronic contribution to specific heat $C_{\text{E}}^{\text{e}}$, although amorphous alloys do not have long-range order, the single electron approximation theory is still applicable (Huang 1988), so the $C_{\text{E}}^{\text{e}}$ is also expressed as $C_{\text{E}}^{\text{e}} = \frac{1}{3}\pi^{2} k_{\text{B}}^{2} N(E_{\text{F}}^{0} )T$. In spite of this, it has been verified that the *C*_p1_ of amorphous alloys cannot be described accurately only by the phonon and the electronic contributions for amorphous alloys (Wang et al. 2011; Tang et al. 2005), this theory needs some improvement. Meanwhile, the existence of random arrangement of atoms is considered to affect the low temperature specific heat *C*_p1_ for amorphous alloy, which means an extra contribution should be included, thus, from the view of disordered microstructure, the random arrangement of atoms has contributions to the *C*_p1_, defined as $C_{\text{E}}^{\text{p}}$. Previous report (Kittel 2005) has pointed out that the $C_{\text{E}}^{\text{p}}$ originates from low-energy excitation, which is related to two-level scattering caused by random arrangement of atoms, and the low-energy excitation is also successfully explained using the two-level scattering model (Vladar and Zawadowski 1983; Cochrane et al. 1975). Therefore, $C_{\text{E}}^{\text{p}}$ is written as $C_{\text{E}}^{\text{p}} = \frac{{2k_{\text{B}}^{2} }}{{3\Delta_{0} }}T$, where $\Delta_{0}$ is the maximum difference between the two energy minimum points. Thus, the low temperature specific heat for amorphous alloy can be expressed as:

2 $$\begin{aligned} C_{\text{p1}} & = C_{\text{E}}^{\text{e}} + C_{\text{D1}} + C_{\text{E}}^{\text{p}} = \frac{1}{3}\pi^{2} k_{\text{B}}^{2} N(E_{\text{F}}^{0} )T + \frac{{12\pi^{4} }}{5}R\left( {\frac{{k_{\text{B}} }}{{\hbar w_{\text{m}} }}} \right)^{3} T^{3} + \frac{{2k_{\text{B}}^{2} }}{{3\Delta_{0} }}T \\ = \left[ {\frac{1}{3}\pi^{2} k_{\text{B}}^{2} N(E_{\text{F}}^{0} ) + \frac{{2k_{\text{B}}^{2} }}{{3\Delta_{0} }}} \right]T + \frac{{12\pi^{4} }}{5}R\left( {\frac{{k_{\text{B}} }}{{\hbar w_{\text{m}} }}} \right)^{3} T^{3} \\ \end{aligned}$$

Thus, Eq. (2) can be further simplified as:

3 $$C_{\text{p1}} = C_{\text{E1}} + C_{\text{D1}} = \gamma_{\text{a}} T + \delta_{\text{a}} T^{3}$$

where $C_{E1} (C_{E1} = C_{\text{E}}^{\text{e}} + C_{\text{E}}^{\text{p}} )$ is the electronic specific heat; $\gamma_{\text{a}} = \frac{1}{3}\pi^{2} k_{\text{B}}^{2} N(E_{\text{F}}^{0} ) + \frac{{2k_{\text{B}}^{2} }}{{3\Delta_{0} }}$; $\delta_{\text{a}} = \frac{{12\pi^{4} }}{5}R\left( {\frac{{k_{\text{B}} }}{{\hbar w_{\text{m}} }}} \right)^{3}$; *k*_B_, $\hbar$, and *R* are fundamental physical constants; and *N*($E_{\text{F}}^{0}$), $\Delta_{0}$, and *w*_m_ are the physical quantities determined by materials, respectively. Here, *γ*_a_ and *δ*_a_ should be invariants for given amorphous alloys.

At low temperature, it is difficult to distinguish the contributions from the electron and from the low-energy excitation (Tang et al. 2005), so *γ*_a_ is usually named as total electronic specific heat coefficient.

By comparing Eqs. (1) and (3), it can be seen that there is a similarity on specific heat equation for amorphous and crystallized alloys, both of them can be represented as a sum of linear electronic term and phonon term, namely:

4 $$C_{p} = \gamma T + \delta T^{3}$$

where *C*_p_ is specific heat; *γ* is electronic specific heat coefficient for amorphous and crystallized alloys; *δ* is phonon specific heat coefficient for amorphous and crystallized alloys. However, although this conclusion is in good agreement with the fitting model, it is important to note that *γ* may have different connotation for amorphous and crystallized alloys.

From Eqs. (2), (3) and (4), we obtain the electronic term in low temperature specific heat for amorphous alloys, expressed as:

5 $$\frac{1}{3}\pi^{2} k_{B}^{2} \left( {1 + \frac{2}{{\pi^{2} \Delta_{0} }}} \right)N(E_{F}^{0} )T = \gamma T$$

where the variation of *γ* can reflect the changes of long-rang disordered atomic arrangement in amorphous structure (Yang et al. 2010).

From Eq. (4), it can be seen that *γ* is determined by *N*($E_{\text{F}}^{0}$) and $\Delta _{0}$ at a certain temperature, and it is also known that $N\left( {E_{\text{F}}^{0} } \right)$ and $\Delta_{0}$ are changed with the material structure. So, the change of *γ* can be discussed from the variations of *N*($E_{\text{F}}^{0}$) and $\Delta_{0}$. On one hand, when *N*($E_{\text{F}}^{0}$) plays a leading role, *γ* is mainly linked to the position of $E_{\text{F}}^{0}$. For amorphous alloys, the $E_{\text{F}}^{0}$ is near the maximum value in the *N*($E_{\text{F}}^{0}$) curve (Cao et al. 2009). When the alloys are annealed from amorphous to crystallized state, *N*($E_{\text{F}}^{0}$) decreases due to the change of $E_{\text{F}}^{0}$ position (Luborsky 1989; Beck and Guntherodt 1983). For transition metal-metalloid amorphous alloys, Chen et al. (1975) has pointed out that the electrons may transfer from the metalloid elements to fill the “*d*” shells of transition metal elements, causing the change of electronic density of states. In transition metal elements systems, the “*d*” shell has not been filled. From the perspective of energy band theory, when the amorphous alloy is formed, the less overlap of “*d*” orbitals between elements results in a narrow band, however, there are 5 “*d*” orbitals in transition metal element, which can make the band staggered, overlapping, result in the increment of *N*($E_{\text{F}}^{0}$) in “*d*” band (Huang 1988). When the alloy is transformed from amorphous state into crystallized state, the electrons near the Fermi surface can obtain sufficient energy, causing the electrons shift outside the Fermi surface. Thus, it results in the decrease of *N*($E_{\text{F}}^{0}$) in “*d*” band, and *γ* is also reduced. In addition, the volume *V* is normally reduced during the alloy crystallization (Kittel 2005). With the constant number of electrons *N*, it can be drawn from the density of states formula $N\left( {E_{\text{F}}^{0} } \right) = \frac{{3m^{*} N}}{{h^{2} }}\left( {\frac{V}{{3\pi ^{2} N}}}\right)^{2/3}$ (*h* is the Planck’s constant and m* is the effective mass of electrons) (Kittel 2005) that *N*($E_{\text{F}}^{0}$) and its corresponding *γ* decrease. On the other hand, when the amorphous alloy is formed from liquid state by rapid quenching, the oversized cage structure, large voids or enough large free volume may be kept (Hirata et al. 2011), where the densities for most amorphous alloys are about 0.5–3 % less than those for corresponding crystallized alloys. In this kind of structure, some solute atoms are loose or weakly bounded in the interstitial intercluster sites and the vibration of these loose “rattler” atoms shows a higher frequency compared with other atoms in amorphous structure. Therefore, due to the reinforced vibration energy, the probability of the tunneling effect will be increased once the atoms overcome the potential energy barrier, which means a relative decrease of $\Delta_{0}$, and this will cause the increment of *γ*. Meanwhile, due to the existence of oversized cage structure in amorphous alloy, the electronic scattering in the process of transmission is enhanced, and it will result in the increase of m* (m* is effective mass of electrons) (Zhang et al. 2001). Thus, *N*($E_{\text{F}}^{0}$) and its corresponding electronic specific heat coefficient *γ* increase. In conclusion, the increment of electronic density of states at the Fermi level and the vibration frequency of localized loose “rattler” atoms in the oversized cage structure are responsible for the enhancement of *γ*, and result in a larger electronic contribution term for amorphous alloys.

In addition, it can also be seen from Table 1 that phonon specific heat coefficient *δ* is decreased and Debye temperature $\theta_{\text{D}}$ is increased during annealing from the cast to crystallized state, and this phenomenon can be discussed using harmonic vibration model. It is known to all that the atomic vibration frequency *w* = (*k*/*m*)^1/2^ (*k* is the restitution coefficient and *m* is the mass of the oscillator). When alloys are annealed from cast to crystallized state, the *k* and its corresponding *w* become larger, moreover, the corresponding *w*_m_ becomes larger (Huang et al. 2014; Bai et al. 2001). According to the Debye temperature $\theta_{\text{D}} = \hbar w_{\text{m}} /k_{\text{B}}$ and $\delta = \frac{{12\pi ^{4} }}{5} \times \frac{R}{{\theta_{\text{D}}^{3} }}$, $\theta_{\text{D}}$ is increased and *δ* is decreased.

## Conclusions

The low temperature specific heats of (Fe_0.5_Co_0.5_)_72_B_20_Si_4_Nb_4_ amorphous and corresponding crystallized alloys have been investigated. It is demonstrated that the cast state for amorphous alloy has larger specific heat compared with its crystallized state at low temperature. Meanwhile, it is also found that the electronic contribution term for alloy in cast state is larger than that in the crystallized state, and this universal phenomenon is interpreted using the electronic density of states and localized harmonic modes based on the vibrations of loose “rattler” atoms in oversized cage structure. The result has important significance for understanding the effect mechanism before and after crystallization on the electronic specific heat for amorphous alloys at low temperature.
